# Multiplexed immunoassay for a serum autoantibody biomarker panel in diagnostic and prognostic prediction of canine mammary tumors

**DOI:** 10.1080/01652176.2024.2435978

**Published:** 2024-12-06

**Authors:** Chih-Ching Wu, Chia-Yu Chang, Pei-Yi Chou, Xiu-Ya Chan, Chun-Chueh Huang, Youngsen Yang, Hao-Ping Liu

**Affiliations:** a Graduate Institute of Biomedical Sciences, College of Medicine, Chang Gung University, Taoyuan, Taiwan; b Department of Medical Biotechnology and Laboratory Science, College of Medicine, Chang Gung University, Taoyuan, Taiwan; c Department of Otolaryngology-Head & Neck Surgery, Linkou Chang Gung Memorial Hospital, Taoyuan, Taiwan; d Molecular Medicine Research Center, Chang Gung University, Taoyuan, Taiwan; e Department of Veterinary Medicine, College of Veterinary Medicine, National Chung Hsing University, Taichung, Taiwan; f Department of Oncology, Taichung Veterans General Hospital, Taichung, Taiwan; g Biotechnology Center, National Chung Hsing University, Taichung, Taiwan

**Keywords:** Canine mammary tumor, autoantibody, biomarker, serum, multiplexed assay

## Abstract

Canine mammary tumor (CMT) is a prevalent and destructive disease often diagnosed at an advanced stage, leading to poor outcomes. Currently, there is a lack of effective biomarkers for early detection and prognostic prediction of CMT. To improve CMT detection, we established a multiplexed immunoassay using a fluorescence bead-based suspension array system to measure serum levels of autoantibodies against four CMT-associated proteins (AGR2, HAPLN1, IGFBP5, and TYMS) in CMT patients. Our data revealed that serum levels of the four autoantibodies (anti-AGR2, anti-HAPLN1, anti-IGFBP5, and anti-TYMS) were significantly elevated in CMT patients (*n* = 158) compared to healthy individuals (*n* = 39). Notably, serum levels of anti-AGR2, anti-HAPLN1, and anti-TYMS in the dogs with stage I CMT (*n* = 56) were higher than those in the healthy group. Using a marker panel consisting of the four autoantibodies for detecting malignant CMT (*n* = 125) achieved a sensitivity of 50.4% and a specificity of 90%. Furthermore, higher levels of anti-AGR2, anti-HAPLN1, anti-IGFBP5, and anti-TYMS were associated with poorer survival in CMT patients. Collectively, we established a multiplexed immunoassay platform to detect serum autoantibodies and demonstrated that a tailored autoantibody marker panel shows potential clinical applicability for the diagnosis and prognosis of CMT.

## Introduction

Canine mammary tumors (CMTs) are the most prevalent neoplasms, constituting 50-70% of tumors in intact female dogs (Gray et al. 2020; Aupperle-Lellbach et al. 2022). Approximately 50% of CMTs are malignant, with half of these carrying a high risk of metastasis and associated with a poor prognosis. Malignant CMTs predominantly originate from epithelial tissues and exhibit diverse histological subtypes. Among these, simple carcinoma and complex carcinoma are the most common (Vascellari et al. 2016). Simple carcinoma is composed of only one cell type, resembling either luminal epithelial cells or myoepithelial cells, while complex carcinoma comprises a malignant epithelial component and a benign myoepithelial or mesenchymal component (Goldschmidt et al. 2011; Sorenmo et al. 2011; Peña et al. 2013).

Molecular classification of CMTs, adapted from human breast cancer research, is based on the expression of estrogen receptor (ER) and human epidermal growth factor receptor 2 (HER2). This classification potentially divides CMTs into four subtypes: luminal A (ER+/HER2−), luminal B (ER+/HER2+), basal (ER−/HER2− with positive basal markers), and HER2-overexpressing (ER−/HER2+) (Sassi et al. 2010; Zheng et al. 2022). However, this immunohistochemical approach does not distinguish between the epithelial and the myoepithelial components in complex and/or mixed CMT subtypes. Additionally, conflicting data exist regarding HER2-positive tumors and the definition and biological behavior of basal-like carcinomas in dogs (Sassi et al. 2010). Long-term studies show that the risk of CMT-associated mortality within two years of diagnosis ranges from 20% to 45% (Peña et al. 2013). Tumor stage and grade are closely linked to clinical outcomes, whereas molecular and histological subtypes show less association (Chang et al. 2005; Sorenmo et al. 2011; Tavasoly et al. 2013).

Histological examination is the gold-standard technique for CMT diagnosis and is routinely used to assess surgical resection margins and predict local tumor recurrence. Pre-surgical diagnosis of CMTs typically relies on fine needle aspiration cytology (FNAC) or core biopsy. However, FNAC has low diagnostic accuracy in differentiating malignant from benign CMTs (Kuppusamy et al. 2019), and reliable diagnosis based on cytology remains challenging. Although core biopsy offers better diagnostic value than FNAC, it is more invasive and time-consuming. Given that approximately 50% of CMTs are malignant, surgical removal of the affected mammary gland and regional lymph nodes remains the most effective therapeutic option for CMTs. Earlier detection improves prognosis for malignant CMTs, underscoring the need for alternative detection methods that can be combined with FNAC to enhance early diagnosis and improve outcomes for dogs with CMT.

To identify new proteins associated with CMTs, we previously conducted quantitative proteomic analyses comparing malignant CMT tissues with hyperplastic and normal tissues (Wu et al. 2019). This analysis revealed a list of proteins highly expressed in malignant CMT tissues. The four most abundant proteins were selected for further validation: anterior gradient 2 (AGR2), hyaluronan and proteoglycan link protein 1 (HAPLN1), insulin-like growth factor-binding protein 5 (IGFBP5), and thymidylate synthetase (TYMS).

AGR2, a protein disulfide isomerase primarily located in the endoplasmic reticulum, is overexpressed in breast cancers, where it promotes tumor growth and metastasis and is linked to poor prognosis (Li et al. 2015; Maarouf et al. 2022; Yuan et al. 2024). HAPLN1 stabilizes proteoglycan monomers with hyaluronic acid in the extracellular cartilage matrix. It has been shown to play pro-tumorigenic roles in malignant pleural mesothelioma (Ivanova et al. 2009) and participate in signaling networks that lead to cancer stemness, proliferation, and adhesion in early-stage HER2-positive breast cancer (Kim et al. 2022). IGFBP5 is important for cell differentiation and tissue remodeling, contributing to breast cancer pathogenesis and affecting drug responses and clinical outcomes (Dittmer 2022). TYMS, an essential enzyme for producing deoxythymidine monophosphate (a DNA precursor), is found at increased levels in various human cancers and is associated with treatment responses and poor prognosis (Song et al. 2021; Mori et al. 2022).

Proteins overexpressed in tumors can function as tumor-associated antigens (TAAs), which are autologous cellular antigens (or autoantigens) expressed at higher levels or aberrantly localized in cancer cells. A humoral immune response to TAAs occurs in precancerous lesions, resulting in autoantibodies present in the serum before the corresponding TAAs are detectable and before the first clinical signs appear (Chang et al. 2021; Chu et al. 2023, Ouyang et al. 2024). Because antibodies are not subject to proteolysis and remain highly stable in serum, there is growing interest in utilizing TAA-elicited autoantibodies as cancer biomarkers due to their persistence and stability compared to other potential markers (Wu et al. 2014).

We previously established in-house enzyme-linked immunosorbent assays (ELISAs) to measure serum levels of autoantibodies against AGR2 (anti-AGR2), HAPLN1 (anti-HAPLN1), IGFBP5 (anti-IGFBP5), and TYMS (anti-TYMS) in dogs with CMT. Our studies demonstrated that serum levels of these autoantibodies are significantly higher in malignant CMT patients compared with the benign CMT and healthy control groups, indicating their potential for the diagnosis or prognosis of malignant CMTs (Chang et al. 2021; Yuan et al. 2022). These findings highlighted the need to identify a tailored panel of tumor-associated autoantibodies and to develop a simultaneous detection platform to enhance clinical applicability.

In this study, we aimed to develop a multiplexed autoantibody detection platform for clinical use to assess the potential of four CMT-associated autoantibodies (anti-AGR2, anti-HAPLN1, anti-IGFBP5, and anti-TYMS) as diagnostic markers for CMT detection. Our data confirmed that the serum levels of these autoantibodies are significantly increased in dogs with CMTs compared to healthy individuals. The four-autoantibody panel achieved a sensitivity of 50.4% and a specificity of 90% for detecting malignant CMT. Furthermore, higher levels of anti-AGR2, anti-HAPLN1, anti-IGFBP5, and anti-TYMS were associated with worse survival outcomes in CMT dogs.

## Materials and methods

### Signalments and serum collection

Serum samples were collected at the Veterinary Medical Teaching Hospital (VMTH), National Chung Hsing University (NCHU), Taichung, Taiwan, from 2018 to 2022. All experimental procedures and sample collection were approved by the Institutional Animal Care and Use Committee (IACUC) of NCHU (Code: 109-002). Written informed consent was obtained from each dog owner before the experimental procedures. Blood samples were collected from 39 healthy female dogs and 158 CMT dogs before partial or complete mastectomy. This included 33 dogs with benign CMTs and 125 ones with malignant CMTs (Table 1). All blood samples were allowed to clot at room temperature for 30 min, then centrifuged at 2500 × g, 4 °C for 15 min. The resulting supernatants (sera) were immediately treated with a protease inhibitor cocktail (VWP Life Science, Avantor, Radnor, PA, USA), aliquoted into 50 μL volumes, and stored at −80 °C until use. To prevent protein degradation, serum samples undergone more than one freeze-thaw cycle were not used.

**Table 1.** Signalments of dogs enrolled in the analysis of serum autoantibodies.

Characteristics	Control	Benign CMT	Malignant CMT
Case number	39	33	125
No. of female/male	39/0	33/0	124/1
Median/range of age (years)	8/2‒17	8.4/2‒16	10/1‒16
Median/mean of body weight (kg)	13.0/13.4	5.6/9.7	7.0/10.6
No. of dogs spayed	36	9	58
Breed			
Beagle	1	2	4
Bichon Frise	1	0	1
Collie	1	1	2
Chihuahua	3	0	6
Dachshund	1	6	15
Golden Retriever	3	1	7
Maltese	2	6	18
Mixed	15	2	26
Poodle	3	6	16
Pomenarian	1	0	1
Schnauzer	2	0	5
Shiba	1	4	3
Shih-Tzu	1	0	6
Spitz	1	1	2
Yorkshire	1	2	3
Other pedigrees	2	2	10
No. of dogs with CMTs at clinical stage I to V^a^	–	–	56/19/22/16/12

^a^Clinical stage was determined according to the modified WHO classification for domestic animals (Sorenmo et al. 2011).

CMTs were definitely diagnosed through histopathological examination of surgically resected tumor tissues. The excised tumors were fixed in neutral-­buffered 10% formalin and processed using standard histologic techniques. Sections stained with hematoxylin and eosin (H&E) were examined for classification of CMT subtypes, following the established system (Goldschmidt et al. 2011). Grade of malignancy (grades 1 to 3) was assessed based on tubule formation, nuclear pleomorphism, and mitosis (Peña et al. 2013). Malignant CMTs were staged according to the modified World Health Organization (WHO) Tumor-Node-Metastasis (TNM) system (Peña et al. 2013; Tavasoly, et al. 2013), where stages I to III reflect increasing primary tumor size, and stages IV and V indicate regional lymph node involvement and distant metastasis, respectively. Tumor size was recorded as the maximum diameter, and lymph node metastasis was confirmed *via* histopathological examination. Distant metastasis was identified by radiography or necropsy.

### Preparation of recombinant canine proteins

His-tagged recombinant proteins of canine AGR2, HAPLN1, IGFBP5, and TYMS were prepared as previously described (Chang et al. 2021; Yuan et al. 2022). In brief, cDNA derived from CMT tissues or CMT cell lines was used as a template to amplify DNA fragments encoding the full-length canine AGR2 (without the signal peptide), canine HAPLN1, and canine IGFBP5 (without a signal peptide) using polymerase chain reaction (PCR). Forward and reverse primer pairs targeting canine *AGR2* (NCBI Gene ID:482333), *HAPLN1* (NCBI Gene ID: 488921), and *IGFBP5* (NCBI Gene ID: 610316) were employed. The resulting DNA fragments were inserted into the pET-24a(+) expression vector *via* the Nde I and Xho I restriction enzyme sites. The DNA fragment for the full-length canine *TYMS* gene (Gene ID: 607417) was synthesized (Protech, Taiwan) and cloned into the pET-24(+) expression vector *via* the Hind III and Xho I sites. The nucleotide sequences of all constructs were confirmed by automatic DNA sequencing (Tri-I Biotech Inc., Taiwan).

The expression vector for individual recombinant proteins was introduced into *Escherichia coli* (*E. coli*) BL21(DE3) for protein expression, induced by 0.5 mM isopropyl β-d-1-thiogalactopyranoside (IPTG). The recombinant proteins were further purified using the nickel-nitrilotriacetic acid (Ni-NTA) Sepharose^TM^ 6 Fast Flow resins (GE Healthcare, Chicago, IL, USA) and dialyzed with 1× PBS at 4 °C overnight. Protein concentrations were determined using a Bicinchoninic acid (BCA) protein assay kit (Pierce, Thermo Fisher Scientific, Waltham, MA, USA).

### Reagents and antibodies

Bio-Plex COOH (carboxylated polystyrene) beads 19 (Cat. No. 171506019), 28 (Cat. No. 171506028), 81 (Cat. No. 171506081), 89 (Cat. No. 171506089), and 95 (Cat. No. 171506095) were purchased from Bio-Rad Laboratories (Hercules, CA, USA). Goat anti-histidine Tag (anti-His) Ab (1.0 mg/mL; Cat. No. AHP1656) and biotin-conjugated rabbit anti-dog IgG (Cat. No. ab136769) were obtained from Bio-Rad Laboratories and Abcam (Cambridge, UK), respectively. Phycoerythrin-labeled streptavidin (SA-PE) was sourced from Jackson ImmunoResearch Laboratories (Cat. No. 016-110-084, West Grove, PA, USA). N-hydroxysulfosuccinimide (NHS; Cat. No. 24500) and 1-ethyl-3-(3-dimethylaminopropyl) carbodiimide (EDC; Cat. No. 22980) were purchased from Thermo Fisher Scientific (Waltham, MA, USA).

### Establishment of a bead-based immunoassay for autoantibody detection

To establish bead-based assays, His-tagged recombinant proteins of canine AGR2, HAPLN1, IGFBP5, and TYMS were covalently coupled to Bio-Plex COOH beads 95, 89, 81, and 28, respectively, following the standard protocol of Bio-Plex amine coupling kit (Cat. No. 171406001, Bio-Rad Laboratories). Bovine serum albumin (BSA; Cat. No. A2153, Sigma-Aldrich, St. Louis, MO, USA) was also conjugated to Bio-Plex COOH bead 19 for normalizing data.

Briefly, 1.5 × 10^6^ Bio-Plex COOH beads were activated with 80 μL of activation buffer (2-(*N*-morpholino)ethanesulfonic acid, 0.1 M, pH 5.4). Then, 10 μL of activation buffer with NHS (50 mg/mL) and EDC (50 mg/mL) were added. After 20 min at room temperature, the beads were washed twice with 150 μL of phosphate-buffered saline (PBS; pH 7.4) and incubated with 6 μg of the recombinant proteins or BSA for 2 h at room temperature. Finally, the beads were washed with 500 μL of PBS (pH 7.4), blocked with 250 μL of blocking buffer, resuspended, and stored in 500 μL of storage buffer at 4 °C until use. The coupling efficiency of each protein was verified with anti-His antibody using the Bio-Plex 200 system (Cat. No. 171000205, Bio-Rad Laboratories).

### Multiplexed detection of IgG autoantibodies in canine serum samples

The assays were conducted using 96-well filter-­bottom microplates (Cat. No. MSBVN1B50, Merck Millipore, Taipei, Taiwan) in a dark room to avoid light exposure. Recombinant protein-conjugated beads (5000 beads for each protein) were mixed and washed in microplates. Each serum sample was diluted 1:25 with PBS containing 1% BSA (Sigma-Aldrich), added to the microplates, and incubated for one hour at room temperature. After washing, 50 μL of biotin-­conjugated anti-canine IgG (1 μg/mL) in PBS containing 1% BSA was applied. Following a 40-minute incubation, 50 μL of SA-PE (diluted 1:1000) in PBS containing 1% BSA was added. After a 20-minute incubation, fluorescence intensities of bead identities and SA-PE were detected using the Bio-Plex 200 system and the Bio-Plex Manager software version 4.2 (Bio-Rad Laboratories). The median fluorescence intensities (MFI) of beads conjugated with individual recombinant proteins were normalized by the MFI of BSA-conjugated beads for each serum sample.

### Follow-up of CMT patients

For all dogs, follow-up examinations, including thoracic radiography, were recommended every two months for the first six months after surgery. A total of 96 dogs with CMTs were followed up periodically *via* telephone interviews every six months for four and a half years post-surgery. Overall survival time (OST) was calculated from the date of surgery to either the time of death or the last follow-up (if still alive). Dogs that died from unrelated causes, such as accidents or heart disease (*n* = 19), were excluded from the analysis. None of the patients underwent euthanasia due to CMT.

### Statistical analysis

Comparison of serum autoantibody levels between groups was evaluated using a two-sample t-test. Levene’s test was employed to determine the quality of variances between the two groups. The 90th percentile of normalized MFI in the control group was set as the cut-off value to determine the sensitivity and specificity of each autoantibody in the other groups. The receiver operating characteristic (ROC) curve was utilized to evaluate the capability of autoantibodies for discriminating CMT dogs from healthy controls. All analyses were performed using the GraphPad Prism V9 software (GraphPad Inc., San Diego, CA, USA). A *p-*value < 0.05 was considered statistically significant.

## Results

### Establishment of a multiplexed immunoassay for profiling of canine serum autoantibodies

**Figure 1.**
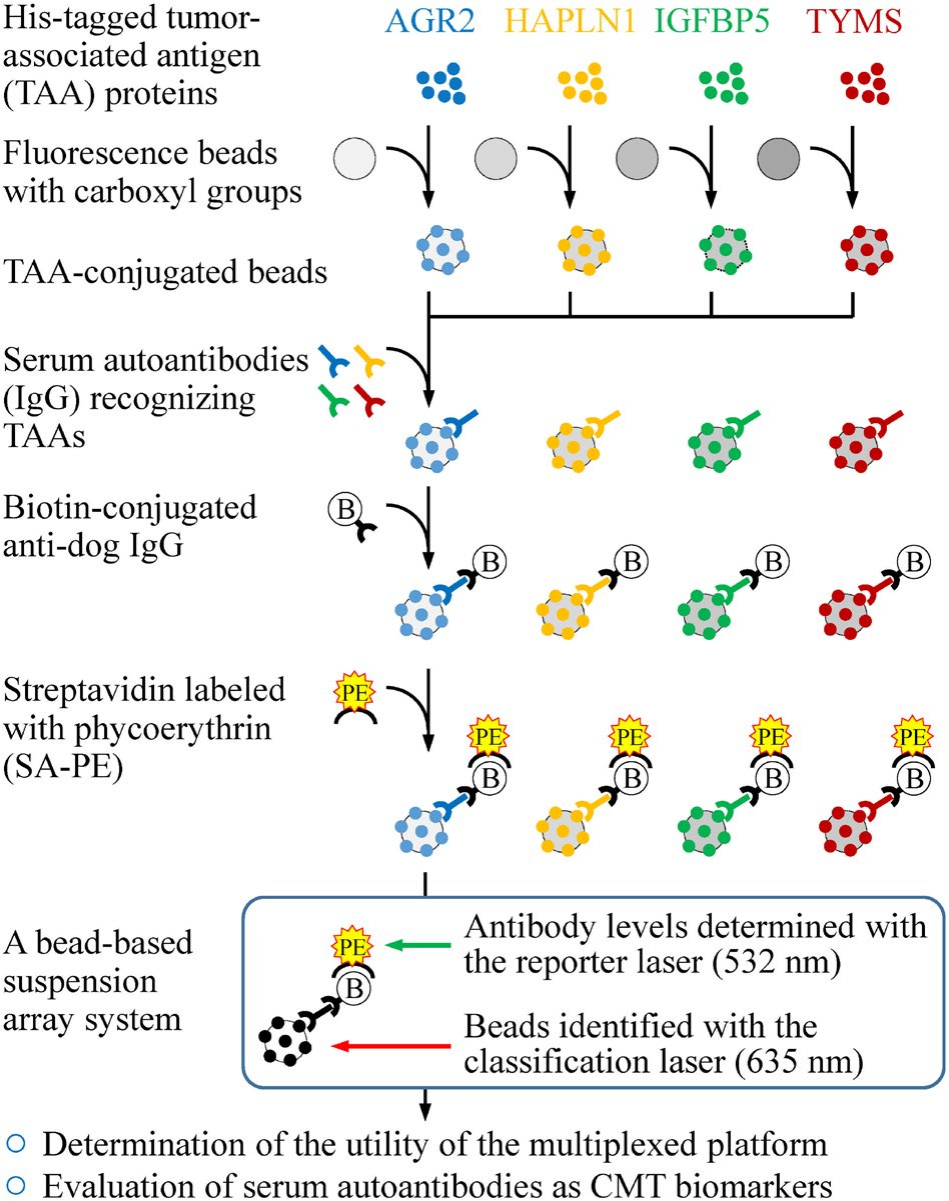
Workflow for establishing a bead-based suspension immunoassay for autoantibody detection in serum samples. Autoantibody detection was conducted using a multiplexed bead-based suspension array system (Bio-plex). To prepare the beads for this system, His-tagged recombinant proteins were covalently conjugated to the COOH beads, each with a unique fluorescence identity. These recombinant protein-­conjugated beads specifically capture the serum IgG that reacts to the recombinant proteins. By addition of a biotin-labeled anti-canine IgG and streptavidin-phycoerythrin (SA-PE), the levels and identities of serum IgG autoantibodies were simultaneously investigated using the Bio-plex system. The “red” classification laser identified the bead identity (and thus the autoantibody type), while the “green” reporter laser assessed the level of the identified autoantibodies. An anti-His antibody was used to evaluate the protein-coupling efficiency. The cross-reactivity of the beads and the accuracy of the multiplex assay are determined using serum samples collected from six canines with CMTs.

We previously identified four proteins (AGR2, HAPLN1, IGFBP5, and TYMS) with elevated levels in malignant CMT tissues (Wu et al. 2019; Yuan et al. 2021). Our further studies demonstrated that these four proteins elicited elevated levels of autoantibodies in the sera of CMT-affected dogs, as measured by in-house ELISAs (Chang et al. 2021; Yuan et al. 2022). Based on these findings, we established a clinically practical multiplexed autoantibody assay using a fluorescence bead-based suspension array, which allows simultaneous measurement of serum levels of the four autoantibodies in a small sample volume (Figure 1).

**Figure 2.**
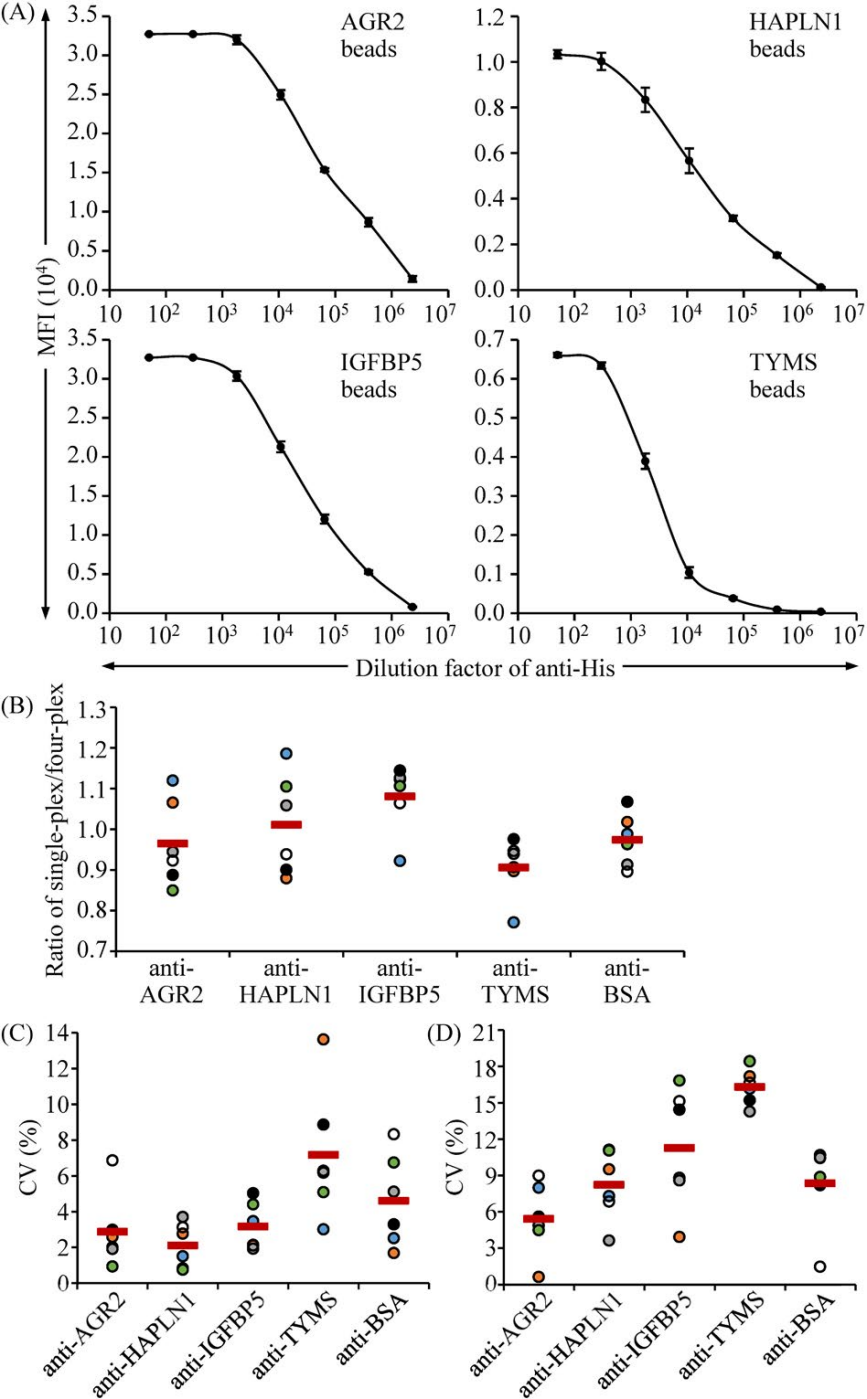
Evaluation of the range and efficiency of autoantibody detection using the established bead-based suspension immunoassay. His-tagged recombinant proteins were conjugated to the COOH beads, each with a unique fluorescence identity. (A) The protein-coupling of individual beads and their effectiveness in antibody detection were verified using an anti-His antibody reacting to the beads coupled with His-tagged AGR2, HAPLN1, IGFBP5, and TYMS. The initial concentration of the anti-His is 20 μg/mL. Data were acquired as the median fluorescence intensity (MFI) and shown as the mean ± SD of MFI. (B) To evaluate the cross-reactivity of the multiplexed immunoassay, serum samples from six canines with CMTs were tested for four types of autoantibodies using individual TAA protein-conjugated beads (single-plex) and a mixture of four TAA-conjugated beads (four-plex). Results are presented as a ratio of autoantibody levels in the single-plex to those in the four-plex for each of the six samples (closed circles), with the mean autoantibody ratio of the six samples indicated by a thick red line. (C) The intra-assay precision of the four-plex immunoassay was determined by measuring autoantibody levels in six samples in triplicate during the same run. (D) The inter-assay precision of the four-plex immunoassay was assessed by measuring autoantibody levels in six samples at three different time points. Results are presented as the coefficient of variation (CV) of autoantibody levels acquired from the three batches of individual samples (closed circles), with the mean CV in the six samples indicated by a thick red line.

To evaluate the efficiency of coupling the His-tagged recombinant proteins to fluorescence beads, the protein-conjugated beads were incubated with a goat anti-His antibody, followed by biotin-labeled anti-goat IgG and SA-PE in the suspension array system. As shown in Figure 2A, the median fluorescence intensity (MFI) of individual protein-conjugated beads reached saturation when incubated with 3.33 μg/mL anti-His antibody. Notably, anti-His antibodies were efficiently and dose-dependently detected using each type of protein-conjugated beads. The dynamic range of anti-His antibody detection spanned three to four orders of magnitude, with the lowest detection limit being 428.67 pg/mL.

### Performance of the multiplexed assay for serum autoantibody detection

To set up a four-plex autoantibody immunoassay, the four types of protein-conjugated beads were mixed with BSA-coupled beads in equal quantities. To evaluate the cross-reactivity in this four-plex assay, the serum levels of individual autoantibodies in six canines with CMTs were measured using both the four-plex and the single-plex assays. As shown in Figure 2B, the ratio of the autoantibody levels determined using the single-plex assay compared to the four-plex setting was close to one (ranging from 0.77 to 1.19) in each case. The mean single-plex/four-plex ratios for anti-AGR2, anti-HPLN1, anti-IGFBP5, and anti-TYMS were 0.97 ± 0.11, 1.01 ± 0.12, 1.08 ± 0.08, and 0.91 ± 0.07, respectively. These results indicate that this four-plex assay effectively achieves multiplexed detection of serum autoantibodies, with limited cross-reactivity.

To evaluate the reliability of this four-plex immunoassay, the detection of four autoantibodies was conducted in triplicates on six serum samples within the same run to assess intra-assay precision. As shown in Figure 2C, the mean coefficient of variation (mean CV) of intra-assays ranged from 2.11% to 7.17% (specifically, 2.88% for anti-AGR2, 2.11% for anti-HAPLN1, 3.17% for anti-IGFBP5, and 7.17% for anti-TYMS). Moreover, individual autoantibody levels were measured in three replicates of the same samples at different time points to evaluate inter-assay precision. The mean CVs of the inter-assays were all below 16% (5.43% for anti-AGR2, 8.24% for anti-HAPLN1, 11.29% for anti-IGFBP5, and 15.32% for anti-TYMS) as shown in Figure 2D. These data demonstrate that this four-plex assay is accurate and efficient for detecting autoantibodies in canine serum samples.

### Multiplexed profiling of four serum autoantibodies in canines with CMTs

**Figure 3.**
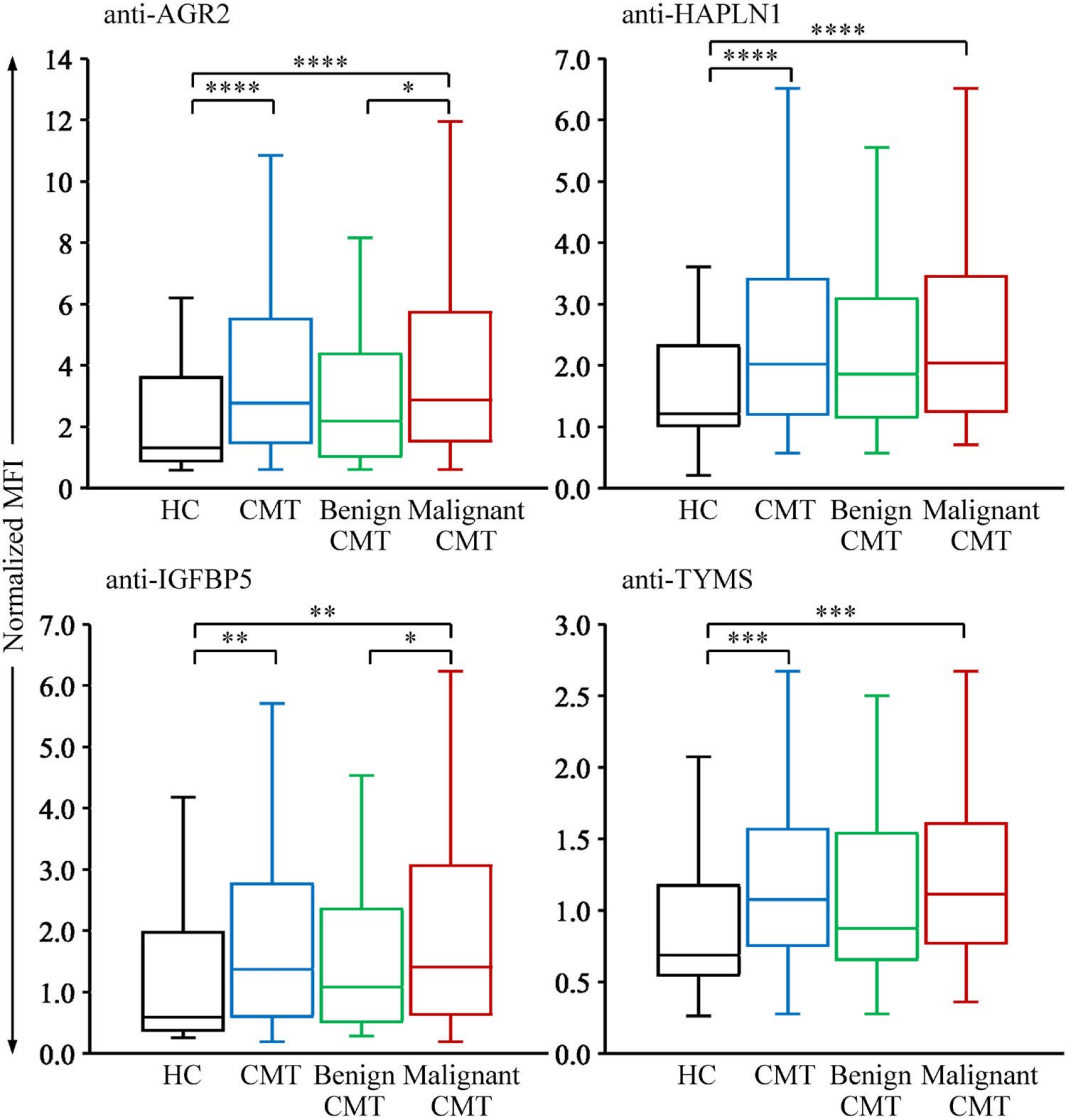
Elevated levels of serum autoantibodies in canines with CMTs. The levels of autoantibodies were detected in serum samples collected from healthy controls (HC; *n* = 39), canines with benign CMT (*n* = 33), and canines with malignant CMT (*n* = 125) using the multiplexed bead-based system. To normalize the data, the MFI of individual autoantibodies in each sample was divided by that of anti-BSA, resulting in normalized MFI values. Serum levels of individual autoantibodies are shown as normalized MFI. The data are displayed as box plots, indicating the upper and lower quartiles, the median value (horizontal line), and the middle 95% distribution (whiskers) of normalized MFI values. *, *p* < 0.05, **, *p* < 0.01, ***, *p* < 0.001, and ****, *p* < 0.0001.

To confirm the association of the four serum autoantibodies with CMT, we assessed their levels in serum samples from 39 healthy controls and 158 canines with CMTs, including 33 with benign tumors and 125 with malignant tumors (Table 1), using the established four-plex assay. As shown in Figure 3, serum levels of all four autoantibodies were higher in the canines with CMTs compared to the healthy group, confirming the association of these autoantibodies with CMT.

Notably, serum levels of the four autoantibodies were significantly elevated in the malignant CMT group compared to the healthy controls (Table 2 and Figure 3). While the autoantibody levels were elevated in the benign CMT group compared with the healthy controls, the difference was not statistically significant. Importantly, anti-AGR2 and anti-IGFBP5 levels were significantly higher in the malignant CMT group compared with the benign CMT group (Figure 3), suggesting their potential as serum biomarkers for CMT malignancy. Further investigation is required to assess the utility of these autoantibodies in predicting CMT malignancy.

**Table 2.** Levels of serum autoantibodies in dogs with CMT.

Groups	*N*	Normalized MFI of autoantibody^a^							
		Anti-AGR2	*p* value^b^	Anti-HAPLN1	*p* value^b^	Anti-IGFBP5	*p* value^b^	Anti-TYMS	*p* value^b^
Healthy control	39	2.23 ± 1.79	–	1.68 ± 1.20	–	1.35 ± 1.49	–	0.89 ± 0.48	–
CMT	158	3.98 ± 3.77	<0.0001	2.79 ± 2.23	<0.0001	2.31 ± 2.70	0.0032	1.41 ± 1.22	0.0001
Benign CMT	33	3.04 ± 2.35	0.1099	2.35 ± 1.59	0.0515	1.67 ± 1.61	0.3865	1.16 ± 0.66	0.0581
Malignant CMT	125	4.23 ± 4.04	<0.0001	2.91 ± 2.37	<0.0001	2.48 ± 2.90	0.0016	1.47 ± 1.32	0.0001
stage I	56	4.10 ± 3.78	0.0018	2.77 ± 2.38	0.0045	2.02 ± 2.03	0.0636	1.30 ± 0.75	0.0015
stages I‒II	75	4.08 ± 3.57	0.0004	2.76 ± 2.23	0.0011	2.26 ± 2.43	0.0143	1.44 ± 1.38	0.0026
stages III‒V	50	4.46 ± 4.68	0.0030	3.12 ± 2.57	0.0008	2.82 ± 3.49	0.0092	1.52 ± 1.23	0.0079
grade 1^c^	33	3.40 ± 3.54	0.0919	2.38 ± 1.83	0.0668	1.74 ± 1.50	0.2752	1.42 ± 1.14	0.0167
grade 2	22	3.59 ± 3.21	0.0778	2.69 ± 2.53	0.0909	2.82 ± 4.44	0.1438	1.14 ± 0.73	0.1630
grade 3	20	4.68 ± 3.53	0.0117	3.36 ± 2.42	0.0108	3.52 ± 3.18	0.0133	2.02 ± 2.31	0.0508
Malignant CMT subtype^c^									
simple	51	3.90 ± 2.66	0.0006	2.84 ± 1.86	0.0006	2.40 ± 2.54	0.0157	1.44 ± 1.41	0.0121
complex	41	4.50 ± 4.82	0.0068	2.90 ± 2.49	0.0070	2.87 ± 3.53	0.0143	1.55 ± 1.37	0.0057
mixed type	20	3.77 ± 3.62	0.0855	2.87 ± 3.10	0.1145	1.77 ± 2.48	0.4852	1.16 ± 0.65	0.1154
other subtype^d^	11	5.50 ± 6.76	0.1428	3.47 ± 2.92	0.0727	2.90 ± 2.91	0.1151	2.04 ± 1.61	0.0410

^a^The autoantibody level is expressed as the median fluorescence intensity (MFI) of tumor-associated autoantibodies divided by that of anti-BSA, resulting in the normalized MFI. The normalized MFI for each autoantibody is presented as the mean ± standard deviation (SD).

^b^The *p* values for comparison between the indicated CMT groups and the healthy control were determined using Student’s *t*-test.

^c^The number of cases with available CMT grade or subtype records is presented.

^d^Other subtypes include myoepithelioma (*n* = 2), ductal or intraductal papillary (*n* = 5), anaplastic (*n* = 2), inflammatory (*n* = 1), and mucinous (*n* = 1) mammary carcinomas.

To assess the correlation between autoantibody levels and CMT grades, we compared the levels of four autoantibodies across the grade 1, 2, and 3 groups with those in the healthy control group. As shown in Table 2, serum levels of anti-AGR2, anti-HAPLN1, and anti-IGFBP5 increased with higher CMT grades. Furthermore, we compared autoantibody levels among the CMT subtypes and the control group. The results showed significant elevation of all four autoantibodies in the simple carcinoma and complex carcinoma subgroups, but no statistically significant differences in the mixed type subgroup.

### Effectiveness of serum autoantibodies for CMT detection using a multiplexed assay

To evaluate the performance of the autoantibodies as serum biomarkers for CMT, we employed receiver operating characteristic (ROC) curve analysis, which determined the effectiveness of the autoantibodies in discriminating canines with CMTs from the healthy controls. As shown in Table 3, the area under the ROC curve (AUC) values of all four autoantibodies in CMT detection were greater than 0.64. Notably, the AUC values of anti-HAPLN1 and anti-TYMS were 0.697 (95% CI: 0.604-0.790) and 0.684 (95% CI: 0.589-0.779), respectively. For detecting malignant CMT, the AUC values of all four autoantibodies were higher than 0.65, with anti-HAPLN1 and anti-TYMS reaching 0.709 (95% CI: 0.615-0.803) and 0.699 (95% CI: 0.602-0.797), respectively.

**Table 3.** Efficacy of using serum autoantibodies as CMT biomarkers.

Autoantibodies	The area under ROC curve (AUC; 95% confidence interval)			
	CMT	Malignant CMT	Malignant CMT at stage I	Malignant CMT at stage I‒II
Anti-AGR2	0.666 (0.573‒0.760)	0.681 (0.587‒0.776)	0.683 (0.573‒0.792)	0.682 (0.579‒0.785)
Anti-HAPLN1	0.697 (0.604‒0.790)	0.709 (0.615‒0.803)	0.699 (0.590‒0.808)	0.702 (0.598‒0.805)
Anti-IGFBP5	0.644 (0.545‒0.744)	0.657 (0.557‒0.757)	0.658 (0.543‒0.774)	0.653 (0.544‒0.762)
Anti-TYMS	0.684 (0.589‒0.779)	0.699 (0.602‒0.797)	0.706 (0.596‒0.816)	0.696 (0.591‒0.802)
Marker panel^a^	0.712 (0.620‒0.803)	0.724 (0.631‒0.817)	0.744 (0.641‒0.839)	0.723 (0.623‒0.818)

^a^A marker panel consisting of anti-AGR2, anti-HAPLN1, anti-IGFBP5, and anti-TYMS was constructed and evaluated using logistic regression analysis.

To further assess the accuracy of these autoantibodies for CMT detection, we calculated their sensitivities (true positive rates) at a given 90% specificity (true negative rate). The sensitivities of the four autoantibodies for CMT detection ranged from 24.1% to 34.8%, and their sensitivities for detecting malignant CMT ranged from 24.8% to 36.0% (Table 4). Among the four autoantibodies, anti-HAPLN1 showed the highest sensitivity for detecting CMT (34.8%) and malignant CMT (36.0%).

**Table 4.** Sensitivities of serum autoantibody biomarkers for the detection of CMT.

Autoantibodies	Cutoff value^a^	Number/Percentage of autoantibody-positive cases				
		Healthy control (*n* = 39)	CMT (*n* = 158)	Benign CMT (*n* = 33)	Malignant CMT (*n* = 125)	Malignant CMT at Stage I‒II (*n* = 75)
Anti-AGR2	4.617	4/10.3%	47/29.7%	7/21.2%	40/32.0%	23/30.7%
Anti-HAPLN1	2.803	4/10.3%	55/34.8%	10/30.3%	45/36.0%	25/33.3%
Anti-IGFBP5	2.747	4/10.3%	40/25.3%	6/18.2%	34/27.2%	20/26.7%
Anti-TYMS	1.612	4/10.3%	38/24.1%	7/21.2%	31/24.8%	18/24.0%
Marker panel^b^	Any 1 Positive	9/23.1%	77/48.7%	14/42.4%	63/50.4%	39/52.0%
	Any 2 Positive	4/10.3%	49/31.0%	8/24.2%	40/32.0%	24/32.0%

^a^At a given specificity of 90%, the normalized MFI of each autoantibody is set as the cutoff value.

^b^A marker panel consists of anti-AGR2, anti-HAPLN1, anti-IGFBP5, and anti-TYMS.

Most importantly, the sensitivity of a four-autoantibody panel was greatly improved, reaching 48.7% for detecting CMT and 50.4% for detecting malignant CMT (Table 4), compared to individual autoantibodies alone. Furthermore, the AUC of the four-autoantibody panel for distinguishing the CMT group from the healthy controls reached 0.712 (95% CI: 0.620-0.803), and the AUC for discriminating malignant CMT from healthy controls reached 0.724 (95% CI: 0.631-0.817). These AUC values are greater than those obtained using individual autoantibodies alone (Table 3). The results demonstrate the potential applicability of the four-autoantibody panel for screening CMT.

### Performance of serum autoantibodies in the detection of early-stage malignant CMTs

**Figure 4.**
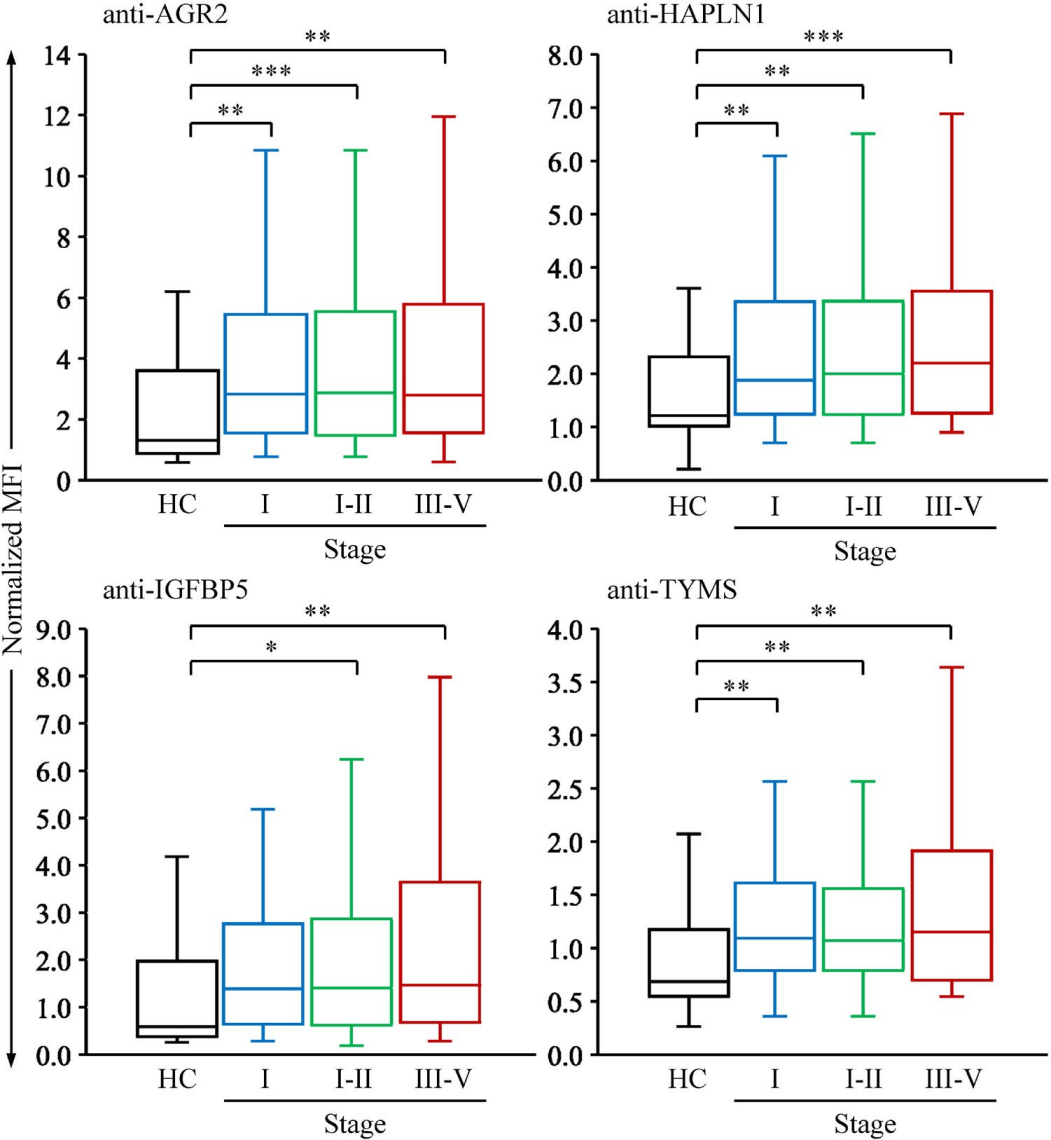
Performance of serum autoantibodies in detecting early-stage malignant CMTs. The levels of autoantibodies were measured in serum samples collected from healthy controls (HC; *n* = 39), canines with malignant CMTs at various stages: I (*n* = 56), I-II (*n* = 75), and III-V (*n* = 50) using the four-plex bead-based system. To normalize the data, the MFI of individual autoantibodies in each sample was divided by that of anti-BSA, resulting in normalized MFI. Serum levels of individual autoantibodies are shown as normalized MFI. The data are shown as box plots, indicating the upper and lower quartiles, the median value (horizontal line), and the middle 95% distribution (whiskers) of normalized MFI values. *, *p* < 0.05, **, *p* < 0.01, ***, *p* < 0.001, and ****, *p* < 0.0001.

To assess the performance of the autoantibodies in detecting early-stage CMT, we evaluated serum autoantibody levels in canines with CMTs at different clinical stages. Compared with the healthy controls, serum levels of the four autoantibodies were significantly elevated in canines with malignant CMTs at clinical stages I-II (Table 2 and Figure 4). Although serum levels of these autoantibodies were elevated in canines with stage III-V CMTs compared to those with stage I-II CMTs, the increase was not statistically significant.

We next analyzed the AUC of the serum autoantibodies for detecting early-stage malignant CMTs. As shown in Table 3, the AUC values of four autoantibodies in distinguishing canines with CMTs at clinical stages I-II from healthy controls ranged from 0.653 to 0.702. At a given specificity of 90%, the sensitivities of the four autoantibodies ranged from 24.0% to 33.3% (Table 4). Notably, using the four-autoantibody panel, we could detect 52.0% (39/75) of canines with CMTs at stages I-II (Table 4).

To further evaluate the efficacy of the four-marker panel in detecting CMT at a very early stage, we analyzed the serum autoantibody levels in canines with stage-I CMTs. As shown in Figure 4, serum levels of anti-AGR2, anti-HAPLN1, and anti-TYMS in the stage-I malignant CMT group were higher compared to the healthy controls. Additionally, the AUC values for distinguishing canines with stage-I CMTs from healthy controls were 0.683 (95% CI: 0.573-0.792) for anti-AGR2, 0.699 (95% CI: 0.590-0.808) for anti-HAPLN1, and 0.706 (95% CI: 0.596-0.816) for anti-TYMS (Table 3). Notably, the AUC for the four-marker panel in screening stage-I malignant CMTs improved to 0.744 (95% CI: 0.641-0.839). These results suggest the potential of these three autoantibodies as serum biomarkers for the early detection of malignant CMTs.

### Correlations of serum autoantibody levels with the survival in canines with CMTs

We further explored the prognostic significance of autoantibody levels in relation to overall survival time (OST). Among the 77 CMT dogs periodically followed, the median OST was 787 days, with a range of 33 − 1597 days.

Next, we assessed the potential of serum autoantibodies to predict survival outcomes in canines with CMTs. The dogs were stratified into high-level and low-level groups based on the median serum autoantibody levels, and survival rates between these groups were analyzed using Kaplan-Meier plots.

**Figure 5.**
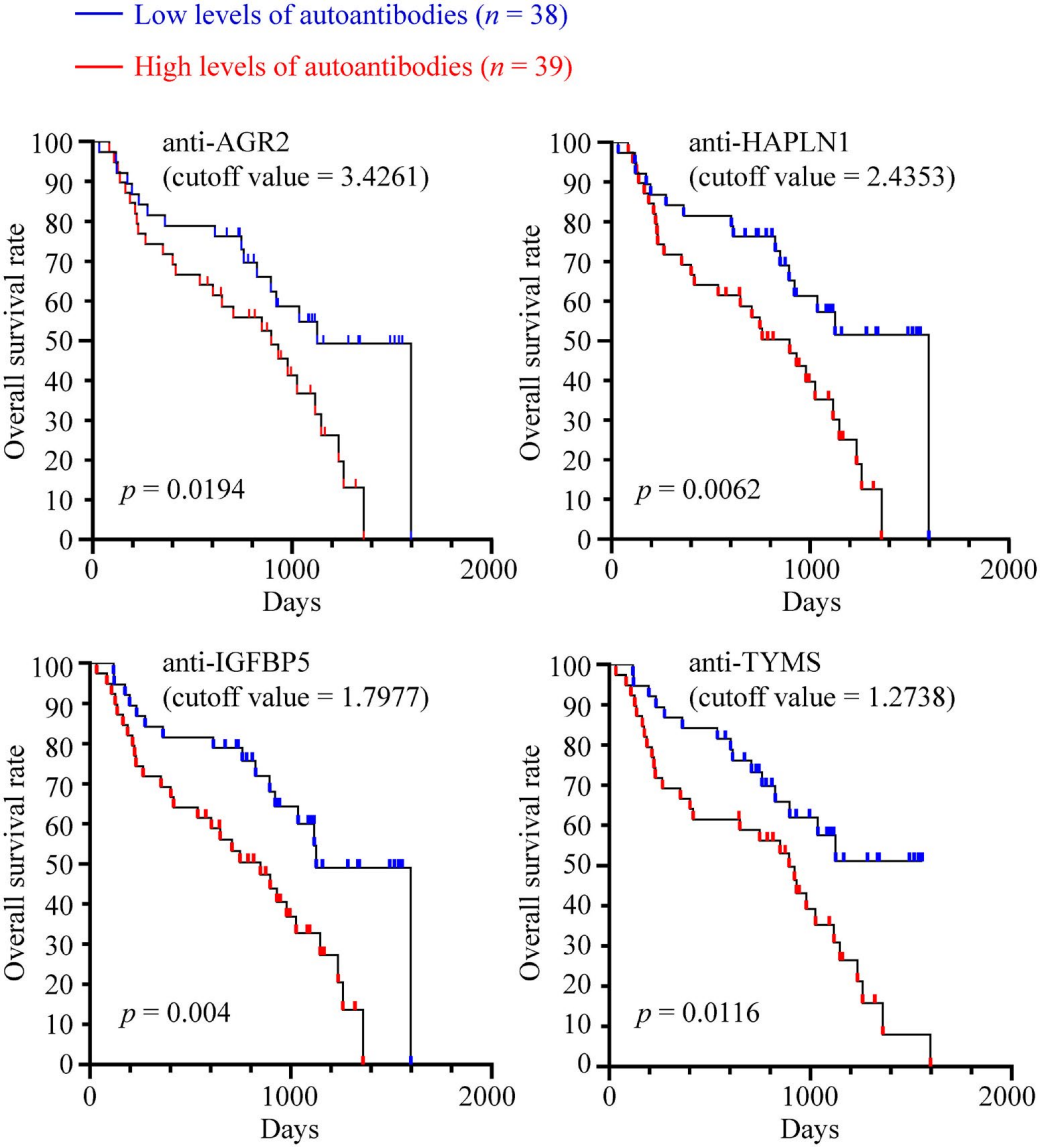
Higher levels of serum autoantibodies correlated with poorer survival rates in canines with CMTs. Kaplan-Meier plots depicting the long-term overall survival of 77 canines with CMTs were analyzed. The canines were stratified using the median serum autoantibody levels as a cutoff value. Statistical significance was determined using log-rank tests.

The results showed that high serum levels of autoantibodies were associated with poorer survival outcomes. As shown in Figure 5, survival rates were significantly lower in the malignant CMT group with high serum levels of anti-AGR2 (*p* = 0.0194), anti-HAPLN1 (*p* = 0.0062), anti-IGFBP5 (*p* = 0.004), and anti-TYMS (*p* = 0.0116) compared to the low-level group.

**Figure 6.**
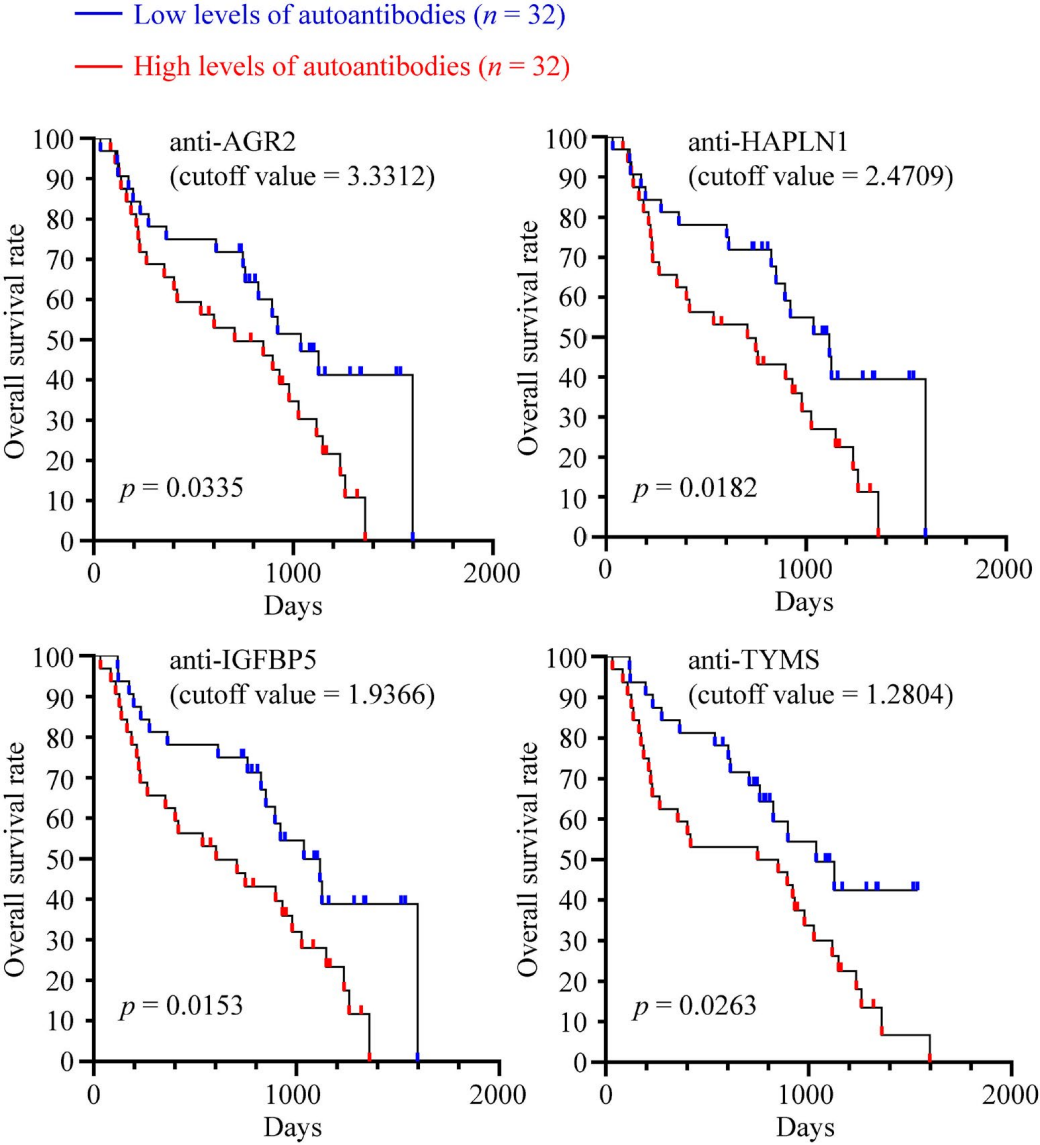
Higher levels of serum autoantibodies correlated with worse survival rates of canines with malignant CMTs. Kaplan-Meier plots illustrated the long-term overall survival of 64 canines with malignant CMTs, stratified by the median serum autoantibody levels as a cutoff value. Statistical significance was assessed using log-rank tests.

For canines with malignant CMTs, high serum levels of anti-AGR2, anti-HAPLN1, anti-IGFBP5, and anti-TYMS were significantly correlated with poorer survival (*p* = 0.0335, 0.0182, 0.0153, and 0.0263, respectively; Figure 6). Specifically, the three-year overall survival rate in the malignant CMT group with high anti-IGFBP5 levels was 32.0%, significantly lower than the 54.9% in the low-level group. Similarly, the three-year overall survival rate in the high anti-HAPLN1 group was 31.7%, compared to 54.8% in the low-level group, and 33.8% in the high anti-TYMS group, compared to 54.7% in the low anti-TYMS group.

These findings suggest that elevated serum levels of these autoantibodies are potential prognostic markers for CMT, indicating poorer survival outcomes.

## Discussion

CMT is a significant global health burden due to its prevalence and severity (Aupperle-Lellbach et al. 2022; Varney et al. 2023; Dhein et al. 2024). Early diagnosis is crucial for improving treatment outcomes. Combining traditional FNAC with biomarker measurement enhances the detection of early-stage CMT (Nosalova et al. 2024). In this study, we established a multiplexed bead-based immunoassay to simultaneously detect serum autoantibodies in canines with CMTs (Figure 1). Compared to single autoantibody assays, this multiplex approach offers robust detection with high sensitivity and specificity, reduced sample usage, and lower costs. As a result, this method shows potential for clinical application in surveying a panel of CMT markers.

Using this multiplexed assay, we demonstrated that the serum levels of autoantibodies against AGR2, HAPLN1, IGFBP5, and TYMS are significantly higher in canines with CMTs than in healthy dogs (Table 2; Figure 3). Notably, their levels are elevated in dogs with malignant CMTs and generally increase along with stage progression (Table 2; Figure 4). At a specificity of 90% for CMT detection, the four autoantibodies analyzed in this study achieve a sensitivity of 24.8% and above for malignant CMT detection (Table 4). This suggests that these autoantibodies may hold early diagnostic potential as screening biomarkers for CMTs. Although the sensitivity of individual autoantibodies is insufficient for reliable detection, the four-marker panel consisting of anti-AGR2, anti-HAPLN1, anti-IGFBP5, and anti-TYMS significantly enhances sensitivity, particularly for detecting early-stage malignant CMTs (sensitivity: 52.0%, specificity: 90%; Table 4). This study highlights the need for further research to identify additional CMT-associated autoantibodies, with the goal of optimizing the panel to improve detection sensitivity.

On the other hand, the higher serum levels of anti-AGR2, anti-HAPLN1, anti-IGFBP5, and anti-TYMS are markedly associated with worse overall survival in canines with CMTs (Figures 5 and 6). Most significantly, the three-year survival rates for malignant CMT dogs with high and low levels of serum anti-IGFBP5 were 32.0% and 54.9%, respectively (*p* = 0.0153; Figure 5), indicating that serum autoantibodies may also have prognostic potential in dogs with CMTs. This finding warrants further confirmation using a larger sample size.

It is presumed that cancer immunosurveillance during tumorigenesis may lead to increased production of cancer-associated autoantibodies (Soussi 2000; Izhak et al. 2010; Al-Hashimi et al. 2017) due to the development of immunogenicity or loss of self-tolerance to self-antigens (Tsunoda et al. 2002; Backes et al. 2011). Conversely, we observed that autoantibody levels in human late-stage cancer patients are relatively lower than in early-stage cancer patients (Wu, et al. 2014; Hsueh et al. 2022; Chu et al. 2023). This outcome may result from late-stage cancer cells evading immunosurveillance through the generation of poorly immunogenic cells and subversion of the immune system (Zitvogel et al. 2006; Dubuisson et al. 2021). In this study, however, the serum levels of CMT-associated autoantibodies between canines with early and advanced stages of CMTs did not show a statistically significant difference (Figure 4). This suggests that the mechanisms of generating cancer-associated autoantibodies are more complex than previously presumed. These findings also indicate varied effects of humoral responses to TAAs. For cancer prognostic applications, it is essential to differentiate between the protective and pathogenic roles of tumor-associated autoantibodies.

In sum, we established a multiplexed autoantibody immunoassay for diagnostic screening and prognostic prediction of CMT. Importantly, we developed a four-autoantibody panel that is effective for detecting malignant CMTs. This immunoassay platform demonstrates significant potential for composing and expanding workable autoantibodies to enhance clinical applicability. Combining this autoantibody panel with traditional FNAC can greatly improve the effectiveness of CMT detection, allowing canines with CMTs to receive tailored treatment regimens and achieve better disease outcomes.
